# Influence of Genetic Variation on Plasma Protein Levels in Older Adults Using a Multi-Analyte Panel

**DOI:** 10.1371/journal.pone.0070269

**Published:** 2013-07-23

**Authors:** Sungeun Kim, Shanker Swaminathan, Mark Inlow, Shannon L. Risacher, Kwangsik Nho, Li Shen, Tatiana M. Foroud, Ronald C. Petersen, Paul S. Aisen, Holly Soares, Jon B. Toledo, Leslie M. Shaw, John Q. Trojanowski, Michael W. Weiner, Brenna C. McDonald, Martin R. Farlow, Bernardino Ghetti, Andrew J. Saykin, for the Alzheimer’s Disease Neuroimaging Initiative (ADNI)

**Affiliations:** 1 Center for Neuroimaging, Department of Radiology and Imaging Sciences, Indiana University School of Medicine, Indianapolis, Indiana, United States of America; 2 Center for Computational Biology and Bioinformatics, Indiana University School of Medicine, Indianapolis, Indiana, United States of America; 3 Department of Medical and Molecular Genetics, Indiana University School of Medicine, Indianapolis, Indiana, United States of America; 4 Department of Mathematics, Rose-Hulman Institute of Technology, Terre Haute, Indiana, United States of America; 5 Department of Neurology, Mayo Clinic, Rochester, Minnesota, United States of America; 6 Department of Neurology, University of California San Diego, San Diego, California, United States of America; 7 Bristol Myers Squibb Co, Wallingford, Connecticut, United States of America; 8 Department of Pathology and Laboratory Medicine, University of Pennsylvania School of Medicine, Philadelphia, Pennsylvania, United States of America; 9 Departments of Radiology, Medicine and Psychiatry, University of California, San Francisco, San Francisco, California, United States of America; 10 Department of Veterans Affairs Medical Center, San Francisco, California, United States of America; 11 Department of Neurology, Indiana University School of Medicine, Indianapolis, Indiana, United States of America; 12 Department of Pathology and Laboratory Medicine, Indiana University School of Medicine, Indianapolis, Indiana, United States of America; Peninsula College of Medicine and Dentistry, United Kingdom

## Abstract

Proteins, widely studied as potential biomarkers, play important roles in numerous physiological functions and diseases. Genetic variation may modulate corresponding protein levels and point to the role of these variants in disease pathophysiology. Effects of individual single nucleotide polymorphisms (SNPs) within a gene were analyzed for corresponding plasma protein levels using genome-wide association study (GWAS) genotype data and proteomic panel data with 132 quality-controlled analytes from 521 Caucasian participants in the Alzheimer’s Disease Neuroimaging Initiative (ADNI) cohort. Linear regression analysis detected 112 significant (Bonferroni threshold *p* = 2.44×10^−5^) associations between 27 analytes and 112 SNPs. 107 out of these 112 associations were tested in the Indiana Memory and Aging Study (IMAS) cohort for replication and 50 associations were replicated at uncorrected *p*<0.05 in the same direction of effect as those in the ADNI. We identified multiple novel associations including the association of rs7517126 with plasma complement factor H-related protein 1 (CFHR1) level at *p*<1.46×10^−60^, accounting for 40 percent of total variation of the protein level. We serendipitously found the association of rs6677604 with the same protein at *p*<9.29×10^−112^. Although these two SNPs were not in the strong linkage disequilibrium, 61 percent of total variation of CFHR1 was accounted for by rs6677604 without additional variation by rs7517126 when both SNPs were tested together. 78 other SNP-protein associations in the ADNI sample exceeded genome-wide significance (5×10^−8^). Our results confirmed previously identified gene-protein associations for interleukin-6 receptor, chemokine CC-4, angiotensin-converting enzyme, and angiotensinogen, although the direction of effect was reversed in some cases. This study is among the first analyses of gene-protein product relationships integrating multiplex-panel proteomics and targeted genes extracted from a GWAS array. With intensive searches taking place for proteomic biomarkers for many diseases, the role of genetic variation takes on new importance and should be considered in interpretation of proteomic results.

## Introduction

Proteins play critical roles in numerous physiological functions and altered protein levels have been associated with disease [1], [2], [3], [4], [5], [6], [7], [8]. Protein analytes are increasingly being employed as disease or treatment biomarkers with recent technological advances enabling simultaneous measurement of multiple proteins. However, progress in biomarker discovery and confirmation is likely to be limited without a better understanding of the genetic basis of protein analyte levels which can be analyzed as continuous phenotypes or quantitative traits (QTs) because variations in genes, which contain the information to encode proteins, may affect the production of proteins leading to altered levels and potentially to disease. Therefore, in genetics research for biomarker discovery and confirmation, an important goal is to robustly identify important functional variants in the genome regardless the difference in assessment method of protein levels. While the emphasis in the search for functional variants is often on the transcriptome or expression, protein analyte measurements can provide another level of assessment of association between genes and their corresponding protein products. Especially when proteins are known to play important roles in disease or treatment, influence of genetic variations associated with the encoded proteins should be considered. In this case, identified functional variants may be used to stratify protein analytes in their interpretation as diagnostic, prognostic, or therapeutic response biomarkers for disease or treatment.

Several studies [9], [10], [11] have investigated the association of single nucleotide polymorphisms (SNPs) with protein levels in humans. The first study [9] used two dimensional difference gel electrophoresis (2D DIGE) technology [12] to measure 544 proteins in 24 human lymphoblastoid cell lines and identified protein expression quantitative trait loci (peQTLs). The second study [10] performed GWAS analysis on the levels of 813 plasma proteins from 96 healthy older individuals, using an aptamer-based proteomic technology [13]. The third study [11] investigated the role of SNPs on levels of 42 serum and plasma proteins measured from 1200 fasting individuals using Enzyme-Linked ImmunoSorbant Assay (ELISA)-based method (R&D systems, HSTA00C). All three of these studies investigated protein QTLs (pQTLs) either from a large number of subjects with a small number of proteins or from a small number of subjects with a large number of proteins. Although some findings in these studies were replicated in a separate report, none of these studies evaluated the genetic effects on two completely independent cohorts as discovery and replication cohorts.

In this study, we used quality-controlled (QC-ed) genome-wide genotype array data and baseline plasma proteomic data by multiplex immunoassay on the Myriad Rules Based Medicine (RBM) Human DiscoveryMAP panel v1.0 using the Luminex100 platform, different from ones used in the previous studies [9], [10], [11], from 521 non-Hispanic Caucasian participants in the Alzheimer’s Disease Neuroimaging Initiative (ADNI) cohort for the discovery phase and from 59 non-Hispanic Caucasian participants in the Indiana Memory and Aging Study (IMAS) cohort for the replication phase. We mainly investigated the effect of individual SNPs (*cis*-effect) within a gene on the corresponding plasma protein level, analyzing 140 gene-protein association pairs. In addition, we examined the percent of total variation in plasma protein levels explained by each SNP (R^2^_SNP_) while accounting for the effect of relevant covariates. This study identified novel associations and replicated some existing findings. Approximately half of the current findings from the ADNI cohort were replicated in the IMAS cohort. The current study also demonstrated that individual SNPs showed remarkable variations in their effects (R^2^_SNP_).

## Materials and Methods

### Ethics Statement

This study was approved by institutional review boards of all participating institutions and written informed consent was obtained from all participants or authorized representatives.

### Alzheimer’s Disease Neuroimaging Initiative (ADNI)

Data used in this study were obtained from the ADNI database (http://adni.loni.ucla.edu/). ADNI was launched in 2004 by the National Institute on Aging, the National Institute of Biomedical Imaging and Bioengineering, the Food and Drug Administration, private pharmaceutical companies, and nonprofit organizations, as a multi-year public-private partnership. The Principal Investigator of this initiative is Michael W. Weiner, MD, VA Medical Center and University of California–San Francisco. ADNI is a multisite longitudinal study, including more than 800 participants, aged 55 to 90, recruited from over 50 sites across the United States and Canada, The participants include approximately 200 cognitively normal older individuals (normal control; NC) to be followed for 3 years, 400 patients diagnosed with mild cognitive impairment (MCI) to be followed for 3 years, and 200 patients diagnosed with early AD to be followed for 2 years at 6- or 12- month intervals. Longitudinal imaging [14], [15], performance on neuropsychological and clinical assessments [16] and biological samples [5], [17] were collected at baseline and at follow-up visits for all or a subset of participants. *APOE* ε2/ε3/ε4 genotype and genome-wide genotyping data [18] are available on the full ADNI sample and longitudinal proteomic data [5] was obtained for 566 selected participants. Further information about ADNI can be found at http://www.adni-info.org.

### Indiana Memory and Aging Study (IMAS)

IMAS is an ongoing longitudinal study, including euthymic older adults with significant cognitive complaints (CC) including memory concerns in the context of cognitive test performance that is within the normal range, patients with early and late MCI (EMCI and LMCI) or mild AD, and age-matched cognitively normal controls (NC) without significant cognitive complaints. Details regarding participant selection criteria and characterization have been described previously [19], [20]. Neuropsychological and clinical assessments, structural and functional MRI, and blood samples were collected for all participants. *APOE* ε2/ε3/ε4 genotype and GWAS data were available on the full IMAS sample. Amyloid PET and longitudinal imaging at follow-up were available on a subset from this ongoing study.

### Participants and Overall Quality Control Procedure

**Figure 1:**
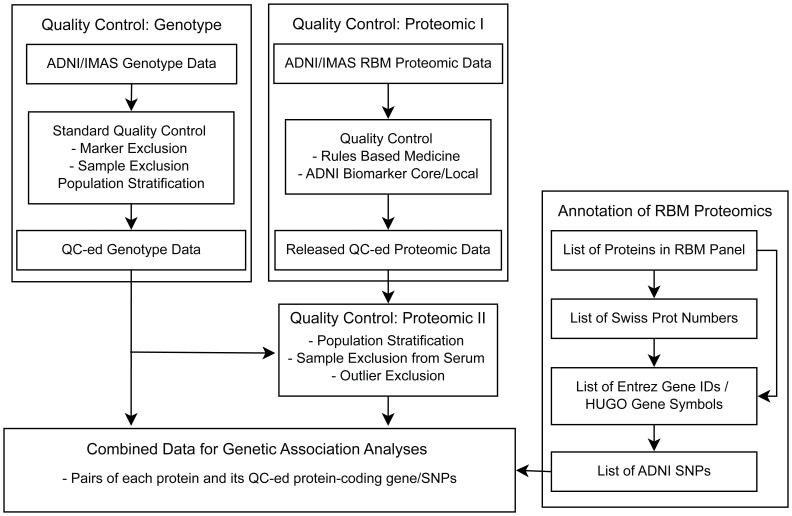
Quality control procedure for genetic and proteomic data.

To reduce the potential bias of population stratification, analyses were restricted to non-Hispanic Caucasian participants from the ADNI (n = 521) (Table 1) and IMAS (n = 59) (Table 2) cohorts. Samples in other racial/ethnic groups were not included in the study because the number of samples in other racial/ethnic groups was relatively small (less than 10%) for genetic analysis in the ADNI and IMAS cohorts. Included participants had GWAS and plasma proteomic data that passed all quality control (QC) procedures (Figure 1) which were similar for the ADNI and IMAS cohorts. Tables 1 and 2 present demographic information for these samples. Data collection and multi-staged QC steps for genotype and proteomic data, each performed separately, are described below and Figure 1 shows the overall flow of this multi-staged QC procedure.

**Table 1:** Sample Characteristics in the ADNI data*.

Characteristics	All	NC	MCI	AD
Number of samples	521	53	360	108
Gender (M/F)	325/196	28/25	234/126	63/45
Baseline Age (years; mean±SD)	75.0±7.36	75.6±5.73	74.9±7.38	75.0±8.01
Education (years; mean±SD)	15.6±2.98	15.8±2.62	15.7±3.01	15.2±3.05
Handedness (R/L)	475/46	49/4	328/32	98/10
*APOE* ε4 status (ε4−/ε4+)	247/274	49/4	163/197	35/73

ADNI: Alzheimer’s Disease Neuroimaging Initiative, NC: normal controls, MCI: mild cognitive impairment, AD: Alzheimer’s disease.

* 521 non-Hispanic Caucasian participants.

**Table 2:** Sample Characteristics in the IMAS data*.

Characteristics	All	NC	CC	EMCI	LMCI	AD
Number of Samples	59	16	18	10	9	6
Gender (M/F)	22/37	5/11	9/9	3/7	4/5	1/5
Age (years; mean±SD)	72.58±7.07	71.79±5.30	71.56±7.86	72.72±8.09	73.49±7.28	76.13±7.77
Education (years; mean±SD)	16.92±2.31	17.38±1.71	17.44±1.89	16.80±2.49	14.89±2.47	17.33±3.27
*APOE* ε4 status (ε4−/ε4+)	39/20	10/6	12/6	9/1	6/3	2/4

IMAS: Indiana Memory and Aging Study, NC: normal controls, CC: cognitive complaints, EMCI: early mild cognitive impairment, LMCI: late mild cognitive impairment, AD: Alzheimer’s disease.

* 59 non-Hispanic Caucasian participants.

### Genotyping and Quality Control

The ADNI protocol for collecting genomic DNA samples from all 818 ADNI participants has been previously described [18], [21]. Genotyping using the Illumina Human610-Quad BeadChip (Illumina, Inc., San Diego, CA), which contains over 600,000 SNP markers, was performed according to the manufacturer’s protocols (Infinium HD Assay; Super Protocol Guide; rev. A, May 2008). A GWAS data set reprocessed in GenomeStudio v2009.1 (Illumina) was downloaded and used for subsequent analyses including all QC procedures.

*APOE* ε2/ε3/ε4 genotypes are defined by two *APOE* SNPs (rs429358 and rs7412) and were separately genotyped at the time of participant enrollment. The two *APOE* SNPs were added to the Illumina genotype data based on the reported *APOE* ε2/ε3/ε4 status prior to assessment of data quality.

All genotype data, including two *APOE* SNPs, underwent standard QC assessment (Figure 1) using PLINK v1.07 [22] (http://pngu.mgh.harvard.edu/~purcell/plink/) [23]. Markers were included using the following criteria: (1) call rate per marker ≥95%, (2) minor allele frequency (MAF) ≥5%, and (3) Hardy-Weinberg Equilibrium (HWE) test *p*≥1.0×10^−6^ in NC participants only. Participants with genotype call rate ≥95% were included and their gender and identity-by-descent were checked to identify genotyping or coding error and to avoid the potential confounding effect due to gender ambiguity or consanguinity such as sibling pairs. In addition, to restrict the present analysis to non-Hispanic Caucasians, 988 founders with known ancestry information from HapMap [24] phase 3 (HapMap3) release 2 were used as reference data in the population stratification step and merged with ADNI samples. In short, ADNI and HapMap3 samples were merged and the multidimensional scaling analysis was performed using PLINK with identity-by-state (IBS) pairwise distance matrix of the merged data. This analysis grouped ADNI and HapMap3 samples in the principle component analysis (PCA) space, allowing us to identify which ADNI samples were grouped with which HapMap3 samples with known ancestry. ADNI participants who were grouped with HapMap3 samples with CEU (Utah residents with ancestry from northern and western Europe from the CEPH collection) or TSI (Toscani in Italia) ancestry and had self-reported race/ethnicity as “non-Hispanic/white” were selected as non-Hispanic Caucasian participants. When ADNI samples were separately grouped from any HapMap3 participants, their self-reported ethnicity and racial information were used in order to be identified as non-Hispanic Caucasian participants. This population stratification analysis identified 749 ADNI participants as non-Hispanic Caucasians and this information was also used for sample selection for the QC procedures of the plasma proteomic data (Figure 1).

The IMAS employed a highly similar protocol to ADNI for collecting blood samples. Genotyping was performed on 85 genomic DNAs using the Illumina HumanOmniExpress BeadChip (Illumina, Inc., San Diego, CA), which contains over 700,000 SNP markers, according to the manufacturer’s protocols (Infinium HD Assay; Super Protocol Guide; Rev. A, May 2008). *APOE* ε2/ε3/ε4 genotyping was separately performed. The two *APOE* SNPs were added to the Illumina genotype data prior to assessment of data quality. All genotype data, including two *APOE* SNPs, underwent the same standard QC assessment (Figure 1) using PLINK v1.07 [22] as the ADNI genotype data. After the final QC step of genotype data, the population stratification, 73 samples were selected as non-Hispanic Caucasians and this information was also used for sample selection for the QC procedures of the plasma proteomic data (Figure 1). For the replication analysis using the IMAS data, if identified SNPs from the discovery phase using the ADNI data were not typed by Illumina HumanOmniExpress, didn’t pass the QC steps or had missing genotypes, those SNPs were imputed using 1000 Genomes reference panel (http://www.1000genomes.org/) following the Enhancing Neuroimaging Genetics through Meta-Analysis 2 (ENIGMA 2) imputation protocol (http://enigma.loni.ucla.edu/wp-content/uploads/2012/07/ENIGMA2_1KGP_v3.pdf [27 July 2012]). Some imputed SNPs were removed based on the following criteria: (1) r^2^<0.5 between imputed and the nearest genotyped SNPs, (2) minor allele frequency <5%.

### Plasma Measurement and Quality Control

Plasma samples were collected from blood for all ADNI participants in the morning before breakfast and after an overnight fast at each visit following the ADNI protocol (for further details see http://www.adni-info.org/Scientists/Pdfs/adniproceduresmanual12.pdf). Briefly, blood samples at each visit were collected into two 10 mL EDTA vacutainer® tubes and centrifuged at room temperature, within one hour of collection, at 3000 rpm (1500 rcf). The plasma fluid was transferred into a labeled 13 mL polypropylene transfer tube, capped, placed upright in dry ice, and shipped to the ADNI Biomarker Core Laboratory at University of Pennsylvania.

A large set of 0.5 mL EDTA plasma samples for a subset of ADNI participants was selected and shipped to Myriad Rules Based Medicine, Inc. (RBM, Austin, TX). Sample selection criteria were explained in the ADNI Biomarker Core Plasma Proteomics Data Primer (http://adni.loni.ucla.edu/wp-content/uploads/2010/11/BC_Plasma_Proteomics_Data_Primer.pdf). A set of 190 protein levels from plasma for each selected individual was measured by multiplex immunoassay on the Human DiscoveryMAP panel v1.0 using the Luminex100 platform by RBM. Additional technical details are available as a white paper from the RBM (http://www.rulesbasedmedicine.com). All QC procedures (Figure 1) by the RBM and ADNI Biomarker Core were previously described in [2], [8] and in the ADNI Biomarker Core Plasma Proteomics Data Primer and statistical analysis plan. In brief, the first QC procedures by the RBM and ADNI Biomarker Core included data transformation if needed, outlier selection (outside ±five standard deviation from mean) and replacement, and imputation of missing/non-measurable values. After these QC steps, 146 out of the 190 proteins in the RBM Human DiscoveryMAP panel for 566 ADNI participants at the baseline visit passed the QC measures and were used in the subsequent QC analyses.

In the second QC step specific to the present study, non-Hispanic Caucasian participants identified from the population stratification analysis of genotype data were selected. RBM (ADNI Biomarker Core Plasma Proteomics Data Primer) reported serum samples as a potential type of specimens for a few datasets (n = 5) and these five datasets were excluded. 521 sample data from plasma at the baseline visit were chosen after these steps. Next, in order to reduce any effect of extreme outlying analyte levels, defined as larger or smaller than four standard deviations from the mean level of each analyte, these extreme values (maximum n = 4 per analyte) were identified and removed from further statistical analyses.

For the IMAS cohort, the ADNI protocol for collecting plasma samples was adopted and all collected plasma samples were stored at the Specimen Storage Facility (SSF) biorepository at Indiana University. A set of 0.5 mL EDTA plasma samples from 68 IMAS participants was selected and shipped to RBM. A set of 185 protein levels (5 assays were discontinued) from plasma for each selected individual was measured by multiplex immunoassay on the RBM Human DiscoveryMAP panel v1.0 that was used for the ADNI samples. The collected proteomic analyte data underwent the initial QC steps, similar to the ADNI Biomarker Core QC steps including outlier detection and replacement, and imputation of missing/non-measurable (“low”) values. Data were not transformed given the limited number of samples in the replication set which did not permit robust distribution analysis. Analytes with less than 10% of missing or non-measurable (“low”) values were imputed (maximum number of imputed values per subject = 3) as follows: missing and non-measurable (“low”) values were imputed to be the mean of the non-missing values and one half of the lowest non-missing value for that analyte, respectively. Finally for each analyte, outliers outside five standard deviations from the mean were assigned the value of the nearest non-outlier point (25 analytes had 1 outlier per analyte). In the second QC step, the QC-ed genotype data were used for selecting non-Hispanic Caucasian participants and one sample was discarded from the analysis due to undetermined diagnosis, resulting in 59 samples with QC-ed proteomic and genotypic data as the replication set. However, further outlier removal outside ±four standard deviation from the mean level of each analyte was not performed due to the limited size of samples.

### Annotation of RBM Proteomics

In order to investigate the genetic influence on each of the 146 protein levels at the baseline visit, we annotated these 146 analytes by identifying their protein-coding genes by mapping the UnitProtKB/Swiss-Prot Accession Numbers of the analytes to the Entrez Gene IDs/HUGO Gene Symbols (Table S1). Then, this list of Gene IDs/Symbols was compared to the list of QC-ed ADNI SNPs. In order to map the QC-ed SNPs to the corresponding genes, we used the Illumina annotation information as an initial mapping step. The annotation information was further tuned using SNP Annotation Tool (http://snp-nexus.org/) [25], [26] based on NCBI36/hg18 and SNP and CNV Annotation Database (http://www.scandb.org/newinterface/about.html). All selected SNPs were inside genes or intergenic within 500 kb margin from gene boundary. If SNPs were intergenic between two genes investigated in the study, SNPs were mapped to the closer gene. Ten proteins were excluded because there were no SNPs within corresponding genes in the QC-ed Illumina Human610-Quad genotype data and four proteins which did not have a UniProtKB/Swiss-Prot Accession Number were excluded. Mapping of proteins to genes was not exactly one-to-one in some cases; seven analytes-Fibrinogen, Follicle-Stimulating Hormone (FSH), Luteinizing Hormone (LH), Thyroid-Stimulating Hormone (TSH), Creatine Kinase-MB (CK-MB), Amphiregulin (AR), Ferritin (FRTN) had more than one UniProtKB/Swiss-Prot Accession Numbers and were mapped to multiple genes (Table S1). Cortisol was included, despite not matching to a UniProtKB/Swiss-Prot Accession Number, because some studies have shown an association with AD, memory performance or cognitive performance [27], [28], [29], [30]. Although the main focus of the present analysis was not the effect of proteomic and genetic data on AD pathology, *CRH* (corticotropin releasing hormone) and *POMC* (proopiomelanocortin) genes, which are indirectly involved in the synthesis/release of cortisol by encoding CRH and Adrenocorticotropic hormone (ACTH), were selected as the corresponding genes to cortisol. After the completion of initial analyses, RBM updated the Swiss-Prot Accession number for Tumor Necrosis Factor receptor 2 (TNFR2) from Q92956 to P20333, changing its protein-coding gene from *TNFRSF14* to *TNFRSF1B*. In addition, the protein name, “complement factor H” has been changed to “complement factor H-related protein 1” together with the Swiss-Prot Accession number from P08603 to Q03591, subsequently changing the protein-coding gene from *CFH* to *CFHR1*. Regarding “complement factor H-related protein 1”, RBM confirmed that the updated annotation should be used for all analyses. Therefore, we repeated the analysis with new sets of SNPs from *TNFRSF1B* and *CFHR1* genes in addition to the originally tested genes (*TNFRSF14* and *CFH*) for TNFR2 and CompFactH, respectively. This slightly increased the number of tests performed. Table S1 contains both of the initial and updated annotation information for TNFR2 and CompFactH.

### Summary of Sample Data and Association

After all QC procedures for genotype and proteomic data, 132 QC-ed analytes (listed under “Tested” column in Table S1) and 1992 QC-ed SNPs belonging to 137 genes for 521 ADNI participants at the baseline visit remained. Tables 1 and 2 present demographic information for the tested sample of 521 ADNI participants and 59 IMAS participants, respectively. In Table S1, a column, “Tested”, indicates which associations were investigated in the ADNI cohort.

### Statistical Analyses

This study investigated the genetic influence on each plasma protein level at the single SNP level within the protein-coding gene. We tested the additive genetic model for each association if the minimum sample size criterion (>10 samples) within each genotype group was satisfied in the ADNI data. When the minimum sample size criterion was not met, the dominant genetic model instead of the additive genetic model was tested. Potential covariates (baseline age, gender, education and handedness) were included in the model if they were significantly associated with the plasma protein level (uncorrected *p*<0.05) using a linear regression analysis in the ADNI data (Table S2).

Because the aim of the present study was to investigate the genetic influences on plasma protein levels, not specific to MCI or AD, baseline diagnosis and Apolipoprotein E (*APOE*) ε4 allele carrier status, the largest known genetic risk factor for sporadic AD, were included in the statistical model as shown below:

#### Model

analyte = constant+SNP+significant covariates+*APOE* ε4 status (ε4−/ε4+)+baseline diagnosis (NC/MCI/AD) for the ADNI cohort/diagnosis (NC/CC/EMCI/LMCI/AD) at the time of plasma collection for the IMAS cohort+error.

One exception was plasma ApoE analyte. Because SNPs within *APOE* gene could be highly correlated with *APOE* ε4 status, resulting in unstable statistical results, *APOE* ε4 status was not included in the model for ApoE level. The statistical model was fitted for each association with additive or dominant genetic model depending on satisfaction of minimum sample size criterion, mentioned above. Analyses were performed using PLINK v1.07. The linear regression function in MATLAB R2009b (The MathWorks, Inc., Natick, MA) was used to test associations of SNPs on the X chromosome in order to separately analyze males and females. For the 132 analytes and 1992 SNPs, a total of 2046 association tests were performed by PLINK or MATLAB in the analyses (see Correction for multiple testing section below) from the ADNI data and identified significant associations from the ADNI sample were investigated using the IMAS data for replication. In the replication analysis, significance of potential covariates (age, gender, and education, but not handedness because all subjects were right-handed) was evaluated with the IMAS samples and the minimum sample size criterion was >10% (6 or more) samples due to the limited size of the replication data set. If this minimum sample size criterion was not satisfied in the IMAS cohort, a dominant genetic model was tested instead of the genetic model, tested in the ADNI cohort.

All analytes for the ADNI sample used in this study were examined for normality of distribution within each diagnostic group by the ADNI Biomarker Core and a large set of the analytes were log-transformed (“LOGTRANS in ADNI” in Table S1). However, these initial procedures did not remove the bi-modal nature or skewness of some analytes over all 521 samples. Although one assumption of linear regression, performed in this study, was the normality of error distribution, the error distribution could change from association to association, depending on the dependent variables (analytes) and its main predictors (SNPs). In order to make it feasible to quantitatively assess the error distributions for 2046 associations, we computed the skewness and kurtosis of analytes and visually assessed the distribution. Therefore, the distribution of analytes over all samples was examined and associations were selected for further scrutiny on the basis of: (1) the absolute value of skewness >2, (2) the absolute value of kurtosis >2, or (3) the subjective assessment of bi-modal distribution from histogram and normal Quantile-Quantile plot. Then, Bootstrap analyses [31] (1000 iterations) were conducted to determine if an analyte with non-normality, e.g., bimodality, resulted in non-normality (Kolmogorov-Smirnov test *p*<0.05) of the sampling distribution of the regression coefficients and, thus, potentially biased p-values. Also, non-parametric analysis of variance (Kruskal-Wallis test [32]) implemented in MATLAB R2009b was performed for these analytes, pre-adjusted for all covariates used in the parametric analyses by using the regression weights. Finally, the p-values from Kruskal-Wallis test were compared to p-values from the linear regression to determine concordance.

For each of the significant associations in the analyses, the percent of total variation explained by each SNP (R^2^_SNP_) from the linear regression model was calculated over all participants while accounting for the effect of other relevant covariates using hierarchical multiple regression as follows:

R^2^_SNP_ = adjusted R^2^ of model with SNP and covariates – adjusted R^2^ of model with covariates.

### Correction for Multiple Testing

In this study, there were 2046 association tests in the ADNI sample between a set of 132 analytes and a set of 1992 SNPs. Therefore, all associations with uncorrected *p*<2.44×10^−5^ ≈ 0.05÷2046 tests (Bonferroni threshold) were considered significant for the ADNI data.

In the IMAS data, due to a limited number of samples (n = 59) and the relatively small number of tests (only significant associations in the ADNI data were tested for replication), no multiple correction methods were applied and any associations with uncorrected *p*<0.05 were considered significant and replicated.

## Results

### Discovery Sample (ADNI)

**Figure 2:**
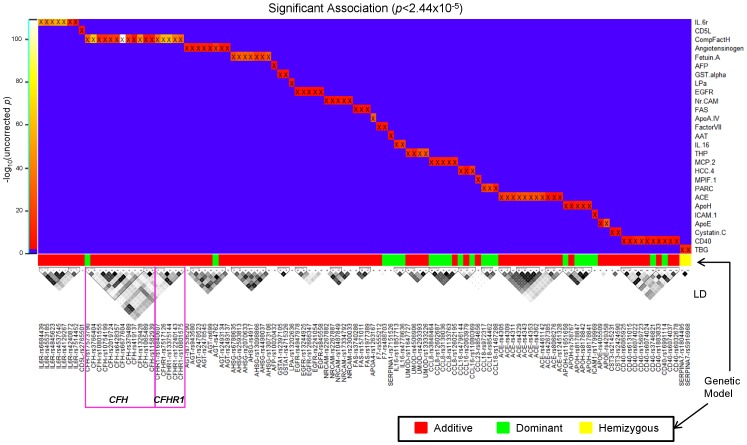
Heatmap of significant associations with genetic models and linkage disequilibrium (LD) in the ADNI cohort. Analyses identified 112 significant (uncorrected *p*<2.44×10^−5^) associations, shown as a heatmap. -log_10_(uncorrected p) was color-coded using the color bar on the left. Below the heatmap (x-axis), tested genetic models and the LD structure within each gene is displayed. Associations of TBG with *SERPINA7* SNPs were significant only for males.

Analyses investigated the effect of individual markers in each gene on corresponding plasma protein levels. Table S3 lists 112 associations between 27 analytes and 112 SNPs in 28 genes at the pre-determined significance level (Bonferroni corrected *p*<0.05, equivalent to uncorrected *p*<2.44×10^−5^ ≈ 0.05÷2046 tests) and all the SNPs had at least 11 samples in each genotype group in the ADNI data. (In Table S1, a column, “Identified”, indicates which associations were identified as significant in the ADNI cohort.) Figure 2 summarizes these 112 associations with tested genetic model. linkage disequilibrium (LD) among SNPs within each gene is shown along the x-axis of the heatmap and -log_10_(uncorrected p) is visualized using the color scale, shown to the left of the heatmap. Figure S1 shows zoomed association results between the 27 identified analytes and SNPs within the 28 corresponding genes (two gene-protein associations between complement factor H-related protein 1 and SNPs in *CFH* and *CFHR1* genes is shown within one panel, CompFactH) using LocusZoom (http://csg.sph.umich.edu/locuszoom/) [33]. Just for visualization purpose in Figure S1, the most significant analyte-SNP pair for each panel was used in order to select the genetic model. All identified SNPs were located in or within a 50 kb margin of each gene. The association of TBG (Thyroxine-Binding Globulin) levels with SNPs in *SERPINA7* (serpin peptidase inhibitor, clade A (alpha-1 antiproteinase, antitrypsin), member 7) gene was significant only for males. The association of rs7517126 with plasma complement factor H-related protein 1 (CFHR1) level at *p*<1.46×10^−60^, accounts for 40 percent of total variation of the protein level. We serendipitously found the association of rs6677604 with the same protein at *p*<9.29×10^−112^. Although these two SNPs were not in the strong LD (highlighted blocks in Figure 2) based on the default algorithm [34] implemented in HaploView [35], 61 percent of total variation of CFHR1 was accounted for by rs6677604 without additional variation by rs7517126 when these two SNPs were tested together in multiple linear regression model. 78 other associations including novel and confirmed associations had uncorrected *p*<5×10^−8^ (generally accepted genome-wide significance level in many GWAS studies [36]). Figure S2 shows the similar pattern of genetic effect within each diagnostic group to the overall pattern for all subjects. This observation in the ADNI sample at least partially supports that the relationship between SNPs and analytes is independent of AD or MCI diagnosis.

749 associations in the ADNI sample were identified as showing non-normality as described in the Materials and Methods section. Thus, the normality of regression coefficients of identified SNPs was examined and all tests, described in the Materials and Methods section, did not identify any significant non-normal cases, reducing the likelihood of significantly biased results due to non-normal distribution of analytes. In addition, the Kruskal-Wallis test [32] found five significant associations that were potentially biased (four associations of FAS and one association of HCC-4 (Chemokine CC-4)) from linear regression analyses. However, according to the normality test of regression coefficient of SNPs from bootstrapping, these five associations from linear regression were less likely to be biased cases due to the non-normal distribution of analytes.

Table 3 shows the percent of total variation explained by the most significantly associated SNP (R^2^_SNP_) within each gene while accounting for the effect of other relevant covariates over all ADNI participants. These SNPs accounted for 3 to 61 percent of the total variation. The associations in Table 3 were sorted in the descending order of R^2^_SNP_. Figure S2 presents scatter plots of the top 12 associations (only rs6677604 for CompFactH) from Table 3 over all ADNI samples and within each diagnostic group. Table S3 shows the complete results of 112 significant associations in the ADNI data.

**Table 3:** List of significant (Bonferroni corrected p<0.05, equivalent to uncorrected p<2.44×10−5) associations in the ADNI cohort.

Analyte	Gene	Chr	SNP	Allele (Major/Minor)	Location*	Function_Class	GM**	R^2^_SNP_	beta	p-value
CompFactH♣	*CFH♣*	1	rs6677604	G/A	196686918	Intron	ADD	0.610	−1567.000	9.29E-112
CompFactH♣	*CFHR1♣*	1	rs7517126	A/G	196840272	Intergenic	ADD	0.397	−1487.458	1.46E-60
IL-6r	*IL6R*	1	rs4129267	C/T	154426264	Intron	ADD	0.386	0.115	1.56E-58
ApoA-IV	*APOA4*	11	rs1263167	A/G	116677723	Intergenic	ADD	0.223	−0.145	6.84E-31
Fetuin-A	*AHSG*	3	rs2070633	C/T	186335941	Intron	ADD	0.219	−0.061	4.50E-31
ACE	*ACE*	17	rs4343	G/A	61566031	cds-synon	ADD	0.205	−0.102	5.20E-28
ApoE***	*APOE*	19	rs429358	T/C	45411941	Missense	ADD	0.196	−0.125	7.27E-30
THP	*UMOD*	16	rs4293393	T/C	20364588	5′ near gene	ADD	0.192	−0.153	2.48E-28
PARC	*CCL18*	17	rs854462	T/C	34386090	Intergenic	DOM	0.148	0.146	9.15E-24
HCC-4	*CCL16*	17	rs11080369	A/C	34305164	Intron	DOM	0.138	−0.218	8.03E-19
TBG♣♣	*SERPINA7*	X	rs1804495	G/T	105278361	missense	HEM	0.137	−0.112	1.24E-12
Angiotensinogen	*AGT*	1	rs4762	C/T	230845977	Missense	DOM	0.136	0.676	4.74E-18
IL-16	*IL16*	15	rs4778636	G/A	81591639	Intron	DOM	0.136	−0.159	5.08E-19
MPIF-1	*CCL23*	17	rs854656	A/C	34345661	5′ near gene	ADD	0.095	−0.069	2.61E-13
MCP-2	*CCL8*	17	rs12602195	A/G	32660149	Intergenic	ADD	0.090	−0.079	2.40E-12
CD40	*CD40*	20	rs1569723	A/C	44742064	Intergenic	ADD	0.088	−0.057	9.61E-14
ApoH	*APOH*	17	rs8178841	C/T	64219197	Intron	DOM	0.071	−68.560	1.08E-10
GST-alpha	*GSTA1*	6	rs4715326	T/C	52657552	Intron	ADD	0.059	−0.135	1.18E-08
Nr-CAM	*NRCAM*	7	rs10487849	T/C	107987085	Intron	ADD	0.058	0.055	1.36E-08
FactorVII	*F7*	13	rs488703	C/T	113770876	Intron	DOM	0.054	−0.098	2.00E-08
LPa	*LPA*	6	rs13202636	T/C	161029728	Intron	ADD	0.048	−0.223	2.27E-07
FAS	*FAS*	10	rs1571011	A/C	90757787	Intron	ADD	0.045	0.039	3.20E-07
ICAM-1	*ICAM1*	19	rs1799969	G/A	10394792	Missense	DOM	0.045	−0.079	1.03E-06
AAT	*SERPINA1*	14	rs7151526	C/A	94863636	Intergenic	DOM	0.043	−0.063	7.44E-07
EGFR	*EGFR*	7	rs13244925	C/A	55192256	Intron	ADD	0.041	0.245	9.12E-07
CD5L	*CD5L*	1	rs2765501	G/A	157804648	Intron	ADD	0.040	0.047	1.52E-06
AFP	*AFP*	4	rs10020432	G/A	74321600	3′ nearGene	ADD	0.032	−0.073	2.26E-05
Cystatin-C	*CST3*	20	rs2424590	T/C	23636980	Intergenic	ADD	0.032	−0.039	4.75E-06

The most significant association of each analyte is listed in the descending order of R^2^_SNP_, which is the fraction of variation accounted for by SNP while adjusting for the effect of covariates. R^2^_SNP_ is defined as follows: R^2^_SNP_ is defined as follows: R^2^_SNP_ = adjusted R^2^ of model with SNP and covariates – adjusted R^2^ of model with covariates.

SNP: Single Nucleotide Polymorphism; ADNI: Alzheimer’s Disease Neuroimaging Initiative; Chr: Chromosome.

* Gene and SNP information are based on the Genome Build 37.3.

** Tested genetic model: Additive (ADD), Dominant (DOM), Hemizygosity (HEM).

*** Statistical model did not include APOE ε4 status due to possible collinearity.

♣Gene-protein pairs in the initial and updated annotation are shown together.

♣♣Association was significant only in males.

### Replication Sample (IMAS)

In the IMAS cohort, one analyte (GST-alpha), associated with two SNPs in the ADNI cohort, didn’t pass proteomic data QC and two SNPs among the 112 SNPs from the ADNI cohort, associated with AAT (Alpha-1-Antitrypsin), and TBG levels (one per each analyte) didn’t pass QC of genotype/imputed data. Also, only one male participant had a minor allele of rs1804495 in *SERPINA7* gene. The association of rs1804495 with TBG for males could not be tested. Therefore, a total of 107 associations were tested in the IMAS cohort. For each association test, if the SNP didn’t satisfy the minimum sample criterion (>5 samples), then a dominant model was tested instead of the genetic model, tested in the ADNI cohort. This analysis replicated 50 associations (Table S4) with uncorrected *p*<0.05 (p-values were not corrected for multiple testing due to limited detection power with the modest sample size of 59). The direction of genetic effect based on beta coefficients and allele coding (major/minor) for these 50 associations was the same as the ADNI results. In Table S1, the last column, “Replicated” indicates which associations were replicated in the IMAS cohort. The most significant association in the ADNI cohort between rs6677604 and complement factor H-related protein 1 was replicated in the IMAS cohort with a dominant genetic model different from the genetic model (additive) in the ADNI cohort due to the minimum sample criterion. For easy comparison between discovery and replication results, Table 4 summarizes the replicated results among all results in Table 3 in the same order as they are listed in Table 3. In case the association from Table 3 was not replicated in the IMAS sample, the most significant association for each analyte in the IMAS sample is listed.

**Table 4:** List of replicated (uncorrected p<0.05) associations in the IMAS cohort.

Analyte	Gene	Chr	SNP	Allele (Major/Minor)	Location*	Function_Class	GM**	R^2^_SNP_	beta	p
CompFactH♣	*CFH♣*	1	rs6677604	G/A	196686918	Intron	DOM	0.280	−1360.286	7.50E-06
CompFactH♣	*CFHR1♣*	1	rs7517126	A/G	196840272	Intergenic	DOM	0.171	−1212.738	6.01E-04
IL-6r	*IL6R*	1	rs4129267	C/T	154426264	Intron	ADD	0.181	4.862	1.69E-04
ApoA-IV	*APOA4*	11	rs1263167	A/G	116677723	Intergenic	DOM	0.066	−4.360	3.42E-02
Fetuin-A	*AHSG*	3	rs2070633	C/T	186335941	Intron	ADD	0.116	−181.751	4.06E-03
ACE	*ACE*	17	rs4343	G/A	61566031	cds-synon	ADD	0.278	−29.226	2.86E-05
PARC	*CCL18*	17	rs854462	A/G	34386090	Intergenic	DOM	0.246	61.739	8.67E-05
HCC-4	*CCL16*	17	rs11080369	A/C	34305164	Intron	DOM	0.183	−2.410	1.01E-03
IL-16	*IL16*	15	rs4778636	G/A	81591639	Intron	DOM	0.123	−74.104	3.83E-03
CD40	*CD40*	20	rs6032678***	C/T	44777295	Intergenic	ADD	0.132	0.089	2.38E-03
ApoH	*APOH*	17	rs8178841	G/A	64219197	Intron	DOM	0.129	−54.359	4.92E-03
FactorVII	*F7*	13	rs488703	G/A	113770876	Intron	DOM	0.047	−118.177	4.20E-02
CD5L	*CD5L*	1	rs2765501	G/A	157804648	Intron	ADD	0.119	597.091	6.46E-03

Among all replicated associations in the IMAS sample, the most significant association of each analyte in the ADNI sample is listed in the same order as they are listed in the Table 3 for easy comparison. In case the most significant SNPs in the ADNI sample was not replicated in the IMAS sample, the most significant association in the IMAS sample is listed. R2SNP, which is the fraction of variation accounted for by SNP while adjusting for the effect of covariates, is defined as follows: R2SNP = adjusted R2 of model with SNP and covariates – adjusted R2 of model with covariates.

SNP: Single Nucleotide Polymorphism; IMAS: Indiana Memory and Aging Study; Chr: Chromosome.

* Gene and SNP information are based on the Genome Build 37.3.

** Tested genetic model: Additive (ADD), Dominant (DOM).

*** SNP with smallest p in the IMAS, but not in the ADNI.

♣Gene-protein pairs in the initial and updated annotation are shown together.

## Discussion

In this study, we were able to identify or confirm the strong influence of genetic variation on circulating plasma protein levels in an older adult population. In some cases this relationship was extraordinarily strong accounting for as much as 61 percent of the variance with *p*<9.29×10^−112^ with 79 other associations exceeding conventional GWAS correction for multiple testing (*p*<5×10^−8^). The biological relevance of the top thirteen gene-protein associations based on R^2^_SNP_ in the ADNI cohort was examined further. Each SNP accounted for 14 to 61 percent of total variation. Among these top 13 gene-protein associations, 9 gene-protein associations were replicated in the IMAS cohort. Two associations, Tamm-Horsfall glycoprotein (THP) and Angiotensinogen, were not replicated in the IMAS cohort. One association, Thyroxine-binding globulin (TBG) with the gene located on the X chromosome, could not be assessed as only a single male participant had the minor allele of the SNP. Association between ApoE level and *APOE* gene was not replicated warranting further investigation.

Among the top 13 associations, a SNP in the *CFHR1* (complement factor H-related 1) gene (rs7517126) showed the very strong influence (R^2^_SNP_) on the plasma level of complement factor H-related protein 1, a complement regulatory protein and a member of complement factor H family. In this study, another SNP in the *CFH* (complement factor H) gene (rs6677604) showed the larger influence (R^2^_SNP_) than rs7517126 although these two SNPs were not in strong LD (Figure 2 and Figure S1). Similar results of these two SNPs on the expression level of *CFHR1* gene were observed in the previous study [37]. It was not clearly explained why rs6677604 has the larger influence on the plasma level of complement factor H-related protein 1 than rs7517126, warranting further investigation. Variations in the *CFH* and *CFHR1* genes have been studied for disease susceptibilities, including age-related macular degeneration [38], [39], dense deposit disease [40], atypical hemolytic-uremic syndrome [41], [42], and systemic lupus erythematosus (SLE) [43]. Plasma complement factor H has been identified as a potential diagnostic biomarker for AD [44]. Interestingly, the SNP with the strongest relationship in this study (rs6677604) has been previously associated with SLE [43].

For interleukin-6 receptor (IL-6r), rs4129267 in the *IL6R* (interleukin 6 receptor) gene had the strongest relationship. The minor allele of the SNP up-regulated the plasma level of IL-6r in the present cohorts. Previous studies reported this association in serum and plasma [10], [11].

Interleukin-16 is a cytokine which functions as a chemoattractant for a variety of CD4+ immune cells and an immunomodulatory cytokine [45]. Two SNPs (rs4778636, rs11857713) in strong LD (pairwise r^2^ = 0.75) influenced plasma level of Interleukin-16. Association of these two SNPs was replicated in the IMAS cohort, but no other studies have reported an association of these SNPs with plasma interleukin-16 level. Association of these SNPs with gene expression in human lymphoblastoid cell lines has been recently reported [10].

Pulmonary and Activation-Regulated Chemokine (PARC) is a small chemokine that belongs to CC chemokine family. Previous studies reported the association of serum PARC with active pulmonary fibrosis in patients with systemic sclerosis [46], and increased plasma level has been observed in childhood acute lymphoblastic leukemia [47] and Gaucher disease [1]. Our study identified three SNPs (rs972317, rs854462, rs1467288) in or near *CCL18* (chemokine (C-C motif) ligand 18 (pulmonary and activation-regulated)) gene, significantly influencing the plasma PARC level in both cohorts, but none of these associations have been previously reported.

Chemokine CC-4 (HCC-4), encoded by *CCL16* (chemokine (C-C motif) ligand 16) gene, is also a small chemokine belonging to CC chemokine family and this chemokine chemoattracts lymphocytes and monocytes but not neutrophils [48]. One SNP out of three identified SNPs in this study (rs2063979) has been associated with visceral leishmaniasis susceptibility in Brazil [49]. The association of rs11080369 and rs2063979 with plasma level of HCC-4 has been previously reported [10]. Although in the present study the effect of rs11080369 was in the same direction, the direction of rs2063979 was opposite to that reported previously indicating that directionality warrants further investigation.

Apolipoprotein E (ApoE) protein plays a role in lipid metabolism, combining with lipids to form lipoproteins. Also, ApoE is a major component of very low-density lipoproteins which remove excess cholesterol from the blood and are known to be bound to high density lipoproteins (HDLs), forming HDL-E, functioning as an inhibitor of agonist induced platelet aggregation [50]. The *APOE* gene encoding ApoE protein is one of the most extensively studied genes, especially for AD susceptibility [51], but also for other disease risk such as cardiovascular mortality [52] and stroke [53]. The relationship between plasma ApoE and AD has been inconsistent [54], [55]. The *APOE* ε4 allele is a well-known risk factor for AD. The rs429358 SNP found to be significantly associated with plasma ApoE in the ADNI cohort is one of two key SNPs determining ε2/ε3/ε4 genotypes. Thus, this SNP not only determines different isoforms of ApoE but it also influences the overall plasma level of ApoE in the ADNI cohort. There was no interaction effect between rs429358 and baseline diagnosis on plasma ApoE at uncorrected *p*<0.05 in an additional analysis. The relationship among rs429358, plasma ApoE levels, and AD should be further investigated using isoform-specific plasma ApoE levels as the platform for measuring plasma ApoE levels did not the measure levels of their specific isoforms.

Apolipoprotein A-IV (ApoA-IV) is another apolipoprotein in plasma that is involved in lipid metabolism. Previous studies have reported an association of ApoA-IV with AD, but the findings are inconsistent [56], [57]. The significant effect of rs1263167 on the plasma level of ApoA-IV was replicated in the IMAS cohort, but has not yet been reported in other studies. One study found the serum level of ApoA-IV to be up-regulated in AD patients [57] and another study observed the association of ApoA-IV deficiency with increased Aß deposition [56].

Human renin-angiotensin system (RAS) plays a role in the regulation of blood pressure, and angiotensiongen and angiotensin-converting enzyme (ACE) are a part of the RAS. Several studies showed the association of *ACE* (angiotensin I converting enzyme (peptidyl-dipeptidase A) 1) variants with AD [58], [59] as well as type 2 diabetic nephropathy [60], and cerebral amyloid angiopathy-related lobar intracerebral hemorrhage recurrence [61]. In our study, rs4343 showed the strongest effect (R^2^_SNP_) on the plasma ACE level. Another study [61] identified the association of rs4311 with serum ACE level in control participants and the present study replicated the finding in the same direction of effect. Plasma angiotensinogen levels are highly heritable [62] and previous studies [63], [64] reported an association of rs4762 and plasma angiotensinogen level. Although rs4762 was associated with plasma angiotensinogen level in the ADNI cohort, the direction was opposite to both previous studies in a Mexican population [63], [64]. In addition, another study failed to identify this association in Nigerians [65]. Further investigation on other influencing factors than genetic variation should be conducted to explain the inconsistency.

Fetuin-A is a serum protein, encoded by *AHSG* (alpha-2-HS-glycoprotein), synthesized in liver and secreted into the blood stream. Plasma Fetuin-A level has been associated with cardiovascular disease [66] and *AHSG* variants have been previously associated with AD [67]. The previous [66] studies identified associations of the same SNPs (rs4917, rs2070633) with plasma Fetuin-A level and in the same direction of effect as was observed in the present study.

Tamm-Horsfall glycoprotein (THP) is abundant in urine, and in humans it is encoded by the *UMOD* (uromodulin) gene, which is associated with chronic kidney disease [68], [69] and blood pressure [70]. We identified four SNPs (rs11647727, rs4506906, rs4293393, rs13333226) associated with the plasma THP level in the ADNI cohort although they were not replicated in the IMAS cohort. Among these SNPs, rs13333226 has been previously associated with diastolic blood pressure [70]. The strongest SNP effect in our study (rs4293393) has also been associated with urinary THP concentrations in the same direction of the observed effect [69].

Thyroxine-binding globulin (TBG) is a protein that is involved in the transport of thyroxine and triiodothyronine in human serum [71]. Previous studies [72], [73] investigated the role of polymorphisms within *SERPINA7* (serpin peptidase inhibitor, clade A (alpha-1 antiproteinase, antitrypsin), member 7) gene in relation to inherited TBG defects. We found rs1804495 to be associated with plasma TBG level but only in males in the ADNI sample. This polymorphism is in codon 303 replacing TTG (leucine) with TTT (phenylalanine) and the role of this variant has not been previously reported on TBG defects or plasma level of TBG.

The present study has some limitations that may be informative for future studies. First, both cohorts consisted of older adults including a large portion with MCI, AD or cognitive complaints. Although age, diagnosis and *APOE* genotype were included as covariates, we could not definitively determine the extent to which age and AD risk may have influenced the observed associations. Studies of gene-protein associations in younger and cognitively healthy samples are needed to clarify the generalizability of the present results. Second, although this study included relevant covariates, other factors than those measured and selected for analysis may have influenced the associations we studied. Further investigation of other factors influencing protein levels beyond genetic variation and the current covariates may be important [5]. Third, non-normal distributions may have influenced association statistics. However, this is relatively unlikely because our analyses did not indicate any significant evidence of statistical bias. Fourth, the IMAS replication sample was of modest size, resulting in limited detection power compared to the ADNI cohort. Additional studies with larger sample sizes are needed for confirmation of the observed relationships. Fifth, the genotyping microarray we used shows considerable variation in SNP coverage for the genes of interest, as illustrated in Figure S1. Therefore, some potential influence of genetic variants on protein analyte levels may have been missed due to undersampling of targeted genomic regions. Imputation of SNP data using HapMap or 1000 reference panel can increase the coverage and will be used in the future study. Finally, there might be technical issues with RBM between the discovery and the replication data which were assayed at different times with different antibodies and conditions used in different RBM runs. The technical issues related to assay time/batch differences could have played roles in those that were not replicated and this is also an issue for future validation of candidate analytes. Considerable amount of work to resolve these and other technical issues inherent to the RBM and follow up assays will be required to evaluate the current findings and turn them into research or clinical grade diagnostic assays in the future.

Despite these limitations, the current study identified 112 SNP-protein associations in the ADNI cohort and many (n = 80) of these associations were highly significant relative to generally accepted significance thresholds (<5×10^−8^). Approximately half of the 112 SNP-protein associations identified in the ADNI cohort were replicated in the IMAS cohort. However, some findings in the ADNI cohort which were not replicated in the IMAS cohort were previously reported in other studies and therefore continue to warrant additional investigation.

In conclusion, this study investigated the role of genetic variation, specifically *cis-*effects, on corresponding protein levels. The strong influence of many genes on commonly measured plasma analytes should be considered. This is particularly critical when proteins are known to play an important role in a disease or treatment. In this case, the evaluation of proteins as diagnostic, prognostic or therapeutic response biomarkers may need to be stratified for genetic background. Future studies should examine diagnostic classification after stratification. Our findings should be replicated in additional independent cohorts with larger samples. It is anticipated that future studies will investigate other genetic mechanisms such as *trans-*effects, haplotypes, copy number variation and epistasis, each of which may influence plasma protein levels. Finally, mRNA sequencing and transcriptome analyses of expression and alternative splicing should provide a more complete picture of functional genetic variations, influencing plasma-gene products.

## Supporting Information

Figure S1 **Zoomed view of association results.** All association results between 28 gene-protein pairs from analyses were shown using LocusZoom (http://csg.sph.umich.edu/locuszoom/). In each panel, hg18 and HapMap Phase II CEU were used as Genome build and LD population. The panel for CompFactH shows the association of SNPs within *CFH* and *CFHR1* together. Just for visualization purpose, the genetic mode tested for the most significant protein-SNP pair for each panel was selected and “DOM” represents a dominant genetic model.
(TIF)

Figure S2 **Scatter plots of top 12 associations in** **Table 3** **for the ADNI cohort.** Different colors represent different diagnoses (black - all, red - AD, green - MCI, blue - NC) and horizontal bars are the average protein levels within each group. Protein levels were adjusted for significant covariates (see Table S2) and *APOE* ε4 status except ApoE. For CompFactH, one association with rs6677604 was shown.
(TIF)

Table S1 **Annotation information for the original 190 proteins in RBM Human DiscoveryMap panel (v.1.0).** Three columns, “LOGTRANS”, “Tested”, and “Identified”, indicate if analytes were log-transformed, which associations were investigated, and which associations were identified as significant in the ADNI cohort, respectively. The last column, “Replicated”, indicates which associations were significantly replicated in the IMAS cohort.
(XLSX)

Table S2 **Inclusion of covariates (baseline age, gender, education and handedness) in the model for each analyte in the ADNI cohort.** Only significant (uncorrected *p*<0.05 on linear regression analyses, marked as “YES”) covariates were included in analyses.
(XLSX)

Table S3 **List of all significant (uncorrected p<2.44×10^−5^) associations in the ADNI cohort.** The associations are sorted by chromosome and chromosomal location of each SNP. Percent of total variation explained by individual SNPs (R^2^_SNP_) while adjusting for the effect of covariates is shown. R^2^_SNP_ is defined as follows: R^2^_SNP_ is defined as follows: R^2^_SNP_ = adjusted R^2^ of model with SNP and covariates – adjusted R^2^ of model with covariates.
(XLSX)

Table S4 **List of all replicated (uncorrected p<0.05) associations in the IMAS cohort.** The associations are sorted by chromosome and chromosomal location of each SNP. Percent of total variation explained by individual SNPs (R^2^_SNP_) while adjusting for the effect of covariates is shown. R^2^_SNP_ is defined as follows: R^2^_SNP_ = adjusted R^2^ of model with SNP and covariates – adjusted R^2^ of model with covariates.
(XLSX)

## References

[1] Boot RG, Verhoek M, de Fost M, Hollak CE, Maas M, et al (2004) Marked elevation of the chemokine CCL18/PARC in Gaucher disease: a novel surrogate marker for assessing therapeutic intervention. Blood 103: 33–39. 12969956 10.1182/blood-2003-05-1612

[2] Chen-Plotkin AS, Hu WT, Siderowf A, Weintraub D, Goldmann Gross R, et al (2011) Plasma epidermal growth factor levels predict cognitive decline in Parkinson disease. Ann Neurol 69: 655–663. 21520231 10.1002/ana.22271 PMC3155276

[3] Hu WT, Chen-Plotkin A, Arnold SE, Grossman M, Clark CM, et al (2010) Biomarker discovery for Alzheimer’s disease, frontotemporal lobar degeneration, and Parkinson’s disease. Acta Neuropathol 120: 385–399. 20652578 10.1007/s00401-010-0723-9 PMC2982700

[4] O’Bryant SE, Xiao G, Barber R, Reisch J, Doody R, et al (2010) A serum protein-based algorithm for the detection of Alzheimer disease. Arch Neurol 67: 1077–1081. 20837851 10.1001/archneurol.2010.215 PMC3069805

[5] Toledo JB, Vanderstichele H, Figurski M, Aisen PS, Petersen RC, et al.. (2011) Factors affecting Abeta plasma levels and their utility as biomarkers in ADNI. Acta Neuropathol. 10.1007/s00401-011-0861-8 PMC3299300 21805181

[6] Hu WT, Holtzman DM, Fagan AM, Shaw LM, Perrin R, et al (2012) Plasma multianalyte profiling in mild cognitive impairment and Alzheimer disease. Neurology 79: 897–905. 22855860 10.1212/WNL.0b013e318266fa70 PMC3425844

[7] Johnstone D, Milward EA, Berretta R, Moscato P (2012) Multivariate protein signatures of pre-clinical Alzheimer’s disease in the Alzheimer’s disease neuroimaging initiative (ADNI) plasma proteome dataset. PLoS One 7: e34341. 22485168 10.1371/journal.pone.0034341 PMC3317783

[8] Soares HD, Potter WZ, Pickering E, Kuhn M, Immermann FW, et al.. (2012) Plasma Biomarkers Associated With the Apolipoprotein E Genotype and Alzheimer Disease. Arch Neurol: 1–8. 10.1001/archneurol.2012.1070 PMC3683865 22801723

[9] Garge N, Pan H, Rowland MD, Cargile BJ, Zhang X, et al (2010) Identification of quantitative trait loci underlying proteome variation in human lymphoblastoid cells. Mol Cell Proteomics 9: 1383–1399. 20179311 10.1074/mcp.M900378-MCP200 PMC2938086

[10] Lourdusamy A, Newhouse S, Lunnon K, Proitsi P, Powell J, et al.. (2012) Identification of cis-regulatory variation influencing protein abundance levels in human plasma. Hum Mol Genet. 10.1093/hmg/dds186 PMC6446535 22595970

[11] Melzer D, Perry JR, Hernandez D, Corsi AM, Stevens K, et al (2008) A genome-wide association study identifies protein quantitative trait loci (pQTLs). PLoS Genet 4: e1000072. 18464913 10.1371/journal.pgen.1000072 PMC2362067

[12] Friedman DB, Lilley KS (2008) Optimizing the difference gel electrophoresis (DIGE) technology. Methods Mol Biol 428: 93–124. 18287770 10.1007/978-1-59745-117-8_6

[13] Gold L, Ayers D, Bertino J, Bock C, Bock A, et al (2010) Aptamer-based multiplexed proteomic technology for biomarker discovery. PLoS One 5: e15004. 21165148 10.1371/journal.pone.0015004 PMC3000457

[14] Jack CR Jr, Bernstein MA, Fox NC, Thompson P, Alexander G, et al (2008) The Alzheimer’s Disease Neuroimaging Initiative (ADNI): MRI methods. J Magn Reson Imaging 27: 685–691. 18302232 10.1002/jmri.21049 PMC2544629

[15] Jagust WJ, Bandy D, Chen K, Foster NL, Landau SM, et al (2010) The Alzheimer’s Disease Neuroimaging Initiative positron emission tomography core. Alzheimers Dement 6: 221–229. 20451870 10.1016/j.jalz.2010.03.003 PMC2920531

[16] Petersen RC, Aisen PS, Beckett LA, Donohue MC, Gamst AC, et al (2010) Alzheimer’s Disease Neuroimaging Initiative (ADNI): clinical characterization. Neurology 74: 201–209. 20042704 10.1212/WNL.0b013e3181cb3e25 PMC2809036

[17] Shaw LM, Vanderstichele H, Knapik-Czajka M, Clark CM, Aisen PS, et al (2009) Cerebrospinal fluid biomarker signature in Alzheimer’s disease neuroimaging initiative subjects. Ann Neurol 65: 403–413. 19296504 10.1002/ana.21610 PMC2696350

[18] Saykin AJ, Shen L, Foroud TM, Potkin SG, Swaminathan S, et al (2010) Alzheimer’s Disease Neuroimaging Initiative biomarkers as quantitative phenotypes: Genetics core aims, progress, and plans. Alzheimers Dement 6: 265–273. 20451875 10.1016/j.jalz.2010.03.013 PMC2868595

[19] Saykin AJ, Wishart HA, Rabin LA, Santulli RB, Flashman LA, et al (2006) Older adults with cognitive complaints show brain atrophy similar to that of amnestic MCI. Neurology 67: 834–842. 16966547 10.1212/01.wnl.0000234032.77541.a2 PMC3488276

[20] Risacher SL, Wudunn D, Pepin SM, Magee TR, McDonald BC, et al.. (2012) Visual contrast sensitivity in Alzheimer’s disease, mild cognitive impairment, and older adults with cognitive complaints. Neurobiology of aging. 10.1016/j.neurobiolaging.2012.08.007 PMC3545045 23084085

[21] Potkin SG, Guffanti G, Lakatos A, Turner JA, Kruggel F, et al (2009) Hippocampal atrophy as a quantitative trait in a genome-wide association study identifying novel susceptibility genes for Alzheimer’s disease. PLoS One 4: e6501. 19668339 10.1371/journal.pone.0006501 PMC2719581

[22] Purcell S, Neale B, Todd-Brown K, Thomas L, Ferreira MA, et al (2007) PLINK: a tool set for whole-genome association and population-based linkage analyses. Am J Hum Genet 81: 559–575. 17701901 10.1086/519795 PMC1950838

[23] Shen L, Kim S, Risacher SL, Nho K, Swaminathan S, et al (2010) Whole genome association study of brain-wide imaging phenotypes for identifying quantitative trait loci in MCI and AD: A study of the ADNI cohort. Neuroimage 53: 1051–1063. 20100581 10.1016/j.neuroimage.2010.01.042 PMC2892122

[24] The International HapMap Project. Nature 426: 789–796. 14685227 10.1038/nature02168

[25] Chelala C, Khan A, Lemoine NR (2009) SNPnexus: a web database for functional annotation of newly discovered and public domain single nucleotide polymorphisms. Bioinformatics 25: 655–661. 19098027 10.1093/bioinformatics/btn653 PMC2647830

[26] Dayem Ullah AZ, Lemoine NR, Chelala C (2012) SNPnexus: a web server for functional annotation of novel and publicly known genetic variants (2012 update). Nucleic acids research 40: W65–70. 22544707 10.1093/nar/gks364 PMC3394262

[27] Huang CW, Lui CC, Chang WN, Lu CH, Wang YL, et al (2009) Elevated basal cortisol level predicts lower hippocampal volume and cognitive decline in Alzheimer’s disease. J Clin Neurosci 16: 1283–1286. 19570680 10.1016/j.jocn.2008.12.026

[28] Laske C, Stransky E, Fritsche A, Eschweiler GW, Leyhe T (2009) Inverse association of cortisol serum levels with T-tau, P-tau 181 and P-tau 231 peptide levels and T-tau/Abeta 1–42 ratios in CSF in patients with mild Alzheimer’s disease dementia. Eur Arch Psychiatry Clin Neurosci 259: 80–85. 18806919 10.1007/s00406-008-0838-3

[29] Lei JK (2010) [Change of serum ACTH and cortisol levels in Alzheimer disease and mild cognition impairment]. Zhonghua Yi Xue Za Zhi 90: 2894–2896. 21211392

[30] Souza-Talarico JN, Chaves EC, Lupien SJ, Nitrini R, Caramelli P (2010) Relationship between cortisol levels and memory performance may be modulated by the presence or absence of cognitive impairment: evidence from healthy elderly, mild cognitive impairment and Alzheimer’s disease subjects. J Alzheimers Dis 19: 839–848. 20157240 10.3233/JAD-2010-1282

[31] Manly BFJ (2007) Randomization, bootstrap and Monte Carly methods in biology; Edition T, editor: Chapman & Hall/CRC Press.

[32] Kruskal WH, Wallis WA (1952) Use of ranks in one-criterion variance analysis. Journal of the American Statistical Association 47: 583–621.

[33] Pruim RJ, Welch RP, Sanna S, Teslovich TM, Chines PS, et al (2010) LocusZoom: regional visualization of genome-wide association scan results. Bioinformatics 26: 2336–2337. 20634204 10.1093/bioinformatics/btq419 PMC2935401

[34] Gabriel SB, Schaffner SF, Nguyen H, Moore JM, Roy J, et al (2002) The structure of haplotype blocks in the human genome. Science 296: 2225–2229. 12029063 10.1126/science.1069424

[35] Barrett JC, Fry B, Maller J, Daly MJ (2005) Haploview: analysis and visualization of LD and haplotype maps. Bioinformatics 21: 263–265. 15297300 10.1093/bioinformatics/bth457

[36] Panagiotou OA, Ioannidis JP (2012) What should the genome-wide significance threshold be? Empirical replication of borderline genetic associations. International journal of epidemiology 41: 273–286. 22253303 10.1093/ije/dyr178

[37] Schroder A, Klein K, Winter S, Schwab M, Bonin M, et al (2013) Genomics of ADME gene expression: mapping expression quantitative trait loci relevant for absorption, distribution, metabolism and excretion of drugs in human liver. The pharmacogenomics journal 13: 12–20. 22006096 10.1038/tpj.2011.44 PMC3564008

[38] Leveziel N, Puche N, Zerbib J, Benlian P, Coscas G, et al (2010) [Genetic factors associated with age-related macular degeneration]. Medecine sciences : M/S 26: 509–515. 20510150 10.1051/medsci/2010265509

[39] Martinez-Barricarte R, Recalde S, Fernandez-Robredo P, Millan I, Olavarrieta L, et al (2012) Relevance of complement factor H-related 1 (CFHR1) genotypes in age-related macular degeneration. Investigative ophthalmology & visual science 53: 1087–1094. 22247456 10.1167/iovs.11-8709

[40] Servais A, Noel LH, Roumenina LT, Le Quintrec M, Ngo S, et al.. (2012) Acquired and genetic complement abnormalities play a critical role in dense deposit disease and other C3 glomerulopathies. Kidney Int. 10.1038/ki.2012.63 22456601

[41] Leban N, Abarrategui-Garrido C, Fariza-Requejo E, Aminoso-Carbonero C, Pinto S, et al (2012) Factor H and CFHR1 polymorphisms associated with atypical Haemolytic Uraemic Syndrome (aHUS) are differently expressed in Tunisian and in Caucasian populations. International journal of immunogenetics 39: 110–113. 22136554 10.1111/j.1744-313X.2011.01071.x

[42] Moore I, Strain L, Pappworth I, Kavanagh D, Barlow PN, et al (2010) Association of factor H autoantibodies with deletions of CFHR1, CFHR3, CFHR4, and with mutations in CFH, CFI, CD46, and C3 in patients with atypical hemolytic uremic syndrome. Blood 115: 379–387. 19861685 10.1182/blood-2009-05-221549 PMC2829859

[43] Zhao J, Wu H, Khosravi M, Cui H, Qian X, et al (2011) Association of genetic variants in complement factor H and factor H-related genes with systemic lupus erythematosus susceptibility. PLoS Genet 7: e1002079. 21637784 10.1371/journal.pgen.1002079 PMC3102741

[44] Thambisetty M, Hye A, Foy C, Daly E, Glover A, et al (2008) Proteome-based identification of plasma proteins associated with hippocampal metabolism in early Alzheimer’s disease. J Neurol 255: 1712–1720. 19156487 10.1007/s00415-008-0006-8

[45] Cruikshank WW, Kornfeld H (2000) Center DM (2000) Interleukin-16. J Leukoc Biol 67: 757–766. 10857846 10.1002/jlb.67.6.757

[46] Kodera M, Hasegawa M, Komura K, Yanaba K, Takehara K, et al (2005) Serum pulmonary and activation-regulated chemokine/CCL18 levels in patients with systemic sclerosis: a sensitive indicator of active pulmonary fibrosis. Arthritis Rheum 52: 2889–2896. 16142750 10.1002/art.21257

[47] Struyf S, Schutyser E, Gouwy M, Gijsbers K, Proost P, et al (2003) PARC/CCL18 is a plasma CC chemokine with increased levels in childhood acute lymphoblastic leukemia. Am J Pathol 163: 2065–2075. 14578205 10.1016/S0002-9440(10)63564-X PMC1892433

[48] Youn BS, Zhang S, Broxmeyer HE, Antol K, Fraser MJ Jr, et al (1998) Isolation and characterization of LMC, a novel lymphocyte and monocyte chemoattractant human CC chemokine, with myelosuppressive activity. Biochem Biophys Res Commun 247: 217–222. 9642106 10.1006/bbrc.1998.8762

[49] Jamieson SE, Miller EN, Peacock CS, Fakiola M, Wilson ME, et al (2007) Genome-wide scan for visceral leishmaniasis susceptibility genes in Brazil. Genes Immun 8: 84–90. 17122780 10.1038/sj.gene.6364357 PMC2495017

[50] Desai K, Bruckdorfer KR, Hutton RA, Owen JS (1989) Binding of apoE-rich high density lipoprotein particles by saturable sites on human blood platelets inhibits agonist-induced platelet aggregation. J Lipid Res 30: 831–840. 2794776

[51] Corder EH, Saunders AM, Strittmatter WJ, Schmechel DE, Gaskell PC, et al (1993) Gene dose of apolipoprotein E type 4 allele and the risk of Alzheimer’s disease in late onset families. Science 261: 921–923. 8346443 10.1126/science.8346443

[52] Mooijaart SP, Berbee JF, van Heemst D, Havekes LM, de Craen AJ, et al (2006) ApoE plasma levels and risk of cardiovascular mortality in old age. PLoS Med 3: e176. 16671834 10.1371/journal.pmed.0030176 PMC1457005

[53] van Vliet P, Mooijaart SP, de Craen AJ, Rensen PC, van Heemst D, et al (2007) Plasma levels of apolipoprotein E and risk of stroke in old age. Ann N Y Acad Sci 1100: 140–147. 17460172 10.1196/annals.1395.012

[54] Gupta VB, Laws SM, Villemagne VL, Ames D, Bush AI, et al (2011) Plasma apolipoprotein E and Alzheimer disease risk: the AIBL study of aging. Neurology 76: 1091–1098. 21422459 10.1212/WNL.0b013e318211c352

[55] Taddei K, Clarnette R, Gandy SE, Martins RN (1997) Increased plasma apolipoprotein E (apoE) levels in Alzheimer’s disease. Neurosci Lett 223: 29–32. 9058415 10.1016/s0304-3940(97)13394-8

[56] Cui Y, Huang M, He Y, Zhang S, Luo Y (2011) Genetic ablation of apolipoprotein A-IV accelerates Alzheimer’s disease pathogenesis in a mouse model. Am J Pathol 178: 1298–1308. 21356380 10.1016/j.ajpath.2010.11.057 PMC3070550

[57] Yang MH, Yang YH, Lu CY, Jong SB, Chen LJ, et al (2012) Activity-dependent neuroprotector homeobox protein: A candidate protein identified in serum as diagnostic biomarker for Alzheimer’s disease. J Proteomics 75: 3617–3629. 22554909 10.1016/j.jprot.2012.04.017

[58] Edwards TL, Pericak-Vance M, Gilbert JR, Haines JL, Martin ER, et al (2009) An association analysis of Alzheimer disease candidate genes detects an ancestral risk haplotype clade in ACE and putative multilocus association between ACE, A2M, and LRRTM3. Am J Med Genet B Neuropsychiatr Genet 150B: 721–735. 19105203 10.1002/ajmg.b.30899 PMC2821734

[59] Ning M, Yang Y, Zhang Z, Chen Z, Zhao T, et al.. (2010) Amyloid-beta-Related Genes SORL1 and ACE are Genetically Associated With Risk for Late-onset Alzheimer Disease in the Chinese Population. Alzheimer Dis Assoc Disord. 10.1097/WAD.0b013e3181e6a575 20625269

[60] Ahluwalia TS, Ahuja M, Rai TS, Kohli HS, Bhansali A, et al (2009) ACE variants interact with the RAS pathway to confer risk and protection against type 2 diabetic nephropathy. DNA Cell Biol 28: 141–150. 19108684 10.1089/dna.2008.0810

[61] Domingues-Montanari S, Hernandez-Guillamon M, Fernandez-Cadenas I, Mendioroz M, Boada M, et al.. (2011) ACE variants and risk of intracerebral hemorrhage recurrence in amyloid angiopathy. Neurobiol Aging 32: 551 e513–522. 10.1016/j.neurobiolaging.2010.01.019 20381197

[62] Watkins WS, Rohrwasser A, Peiffer A, Leppert MF, Lalouel JM, et al (2010) AGT genetic variation, plasma AGT, and blood pressure: An analysis of the Utah Genetic Reference Project pedigrees. Am J Hypertens 23: 917–923. 20414195 10.1038/ajh.2010.83

[63] Balam-Ortiz E, Esquivel-Villarreal A, Alfaro-Ruiz L, Carrillo K, Elizalde A, et al (2011) Variants and haplotypes in angiotensinogen gene are associated with plasmatic angiotensinogen level in Mexican population. Am J Med Sci 342: 205–211. 21629041 10.1097/MAJ.0b013e3182121020 PMC3203016

[64] Balam-Ortiz E, Esquivel-Villarreal A, Huerta-Hernandez D, Fernandez-Lopez JC, Alfaro-Ruiz L, et al (2012) Hypercontrols in genotype-phenotype analysis reveal ancestral haplotypes associated with essential hypertension. Hypertension 59: 847–853. 22371359 10.1161/HYPERTENSIONAHA.111.176453 PMC3306465

[65] Rotimi C, Cooper R, Ogunbiyi O, Morrison L, Ladipo M, et al (1997) Hypertension, serum angiotensinogen, and molecular variants of the angiotensinogen gene among Nigerians. Circulation 95: 2348–2350. 9170394 10.1161/01.cir.95.10.2348

[66] Fisher E, Stefan N, Saar K, Drogan D, Schulze MB, et al (2009) Association of AHSG gene polymorphisms with fetuin-A plasma levels and cardiovascular diseases in the EPIC-Potsdam study. Circ Cardiovasc Genet 2: 607–613. 20031641 10.1161/CIRCGENETICS.109.870410

[67] Geroldi D, Minoretti P, Bianchi M, Di Vito C, Reino M, et al (2005) Genetic association of alpha2-Heremans-Schmid glycoprotein polymorphism with late-onset Alzheimer’s disease in Italians. Neurosci Lett 386: 176–178. 16002217 10.1016/j.neulet.2005.06.014

[68] Gudbjartsson DF, Holm H, Indridason OS, Thorleifsson G, Edvardsson V, et al (2010) Association of variants at UMOD with chronic kidney disease and kidney stones-role of age and comorbid diseases. PLoS Genet 6: e1001039. 20686651 10.1371/journal.pgen.1001039 PMC2912386

[69] Kottgen A, Hwang SJ, Larson MG, Van Eyk JE, Fu Q, et al (2010) Uromodulin levels associate with a common UMOD variant and risk for incident CKD. J Am Soc Nephrol 21: 337–344. 19959715 10.1681/ASN.2009070725 PMC2834540

[70] Han J, Chen Y, Liu Y, Liang Y, Wang X, et al.. (2012) Common variants of the UMOD promoter associated with blood pressure in a community-based Chinese cohort. Hypertens Res. 10.1038/hr.2012.51 22592667

[71] Bartalena L (1990) Recent achievements in studies on thyroid hormone-binding proteins. Endocr Rev 11: 47–64. 2108013 10.1210/edrv-11-1-47

[72] Janssen OE, Bertenshaw R, Takeda K, Weiss R, Refetoff S (1992) Molecular basis of inherited thyroxine-binding globulin defects. Trends Endocrinol Metab 3: 49–53. 18407078 10.1016/1043-2760(92)90043-z

[73] Takeda K, Mori Y, Sobieszczyk S, Seo H, Dick M, et al (1989) Sequence of the variant thyroxine-binding globulin of Australian aborigines. Only one of two amino acid replacements is responsible for its altered properties. J Clin Invest 83: 1344–1348. 2495303 10.1172/JCI114021 PMC303827

