# Supplementary figures and images for: Influence of Genetic Variation on Plasma Protein Levels in Older Adults Using a Multi-Analyte Panel
Source: PLoS One. 2013 Jul 23;8(7):e70269. doi: 10.1371/journal.pone.0070269 (PMC3720913; doi:10.1371/journal.pone.0070269)

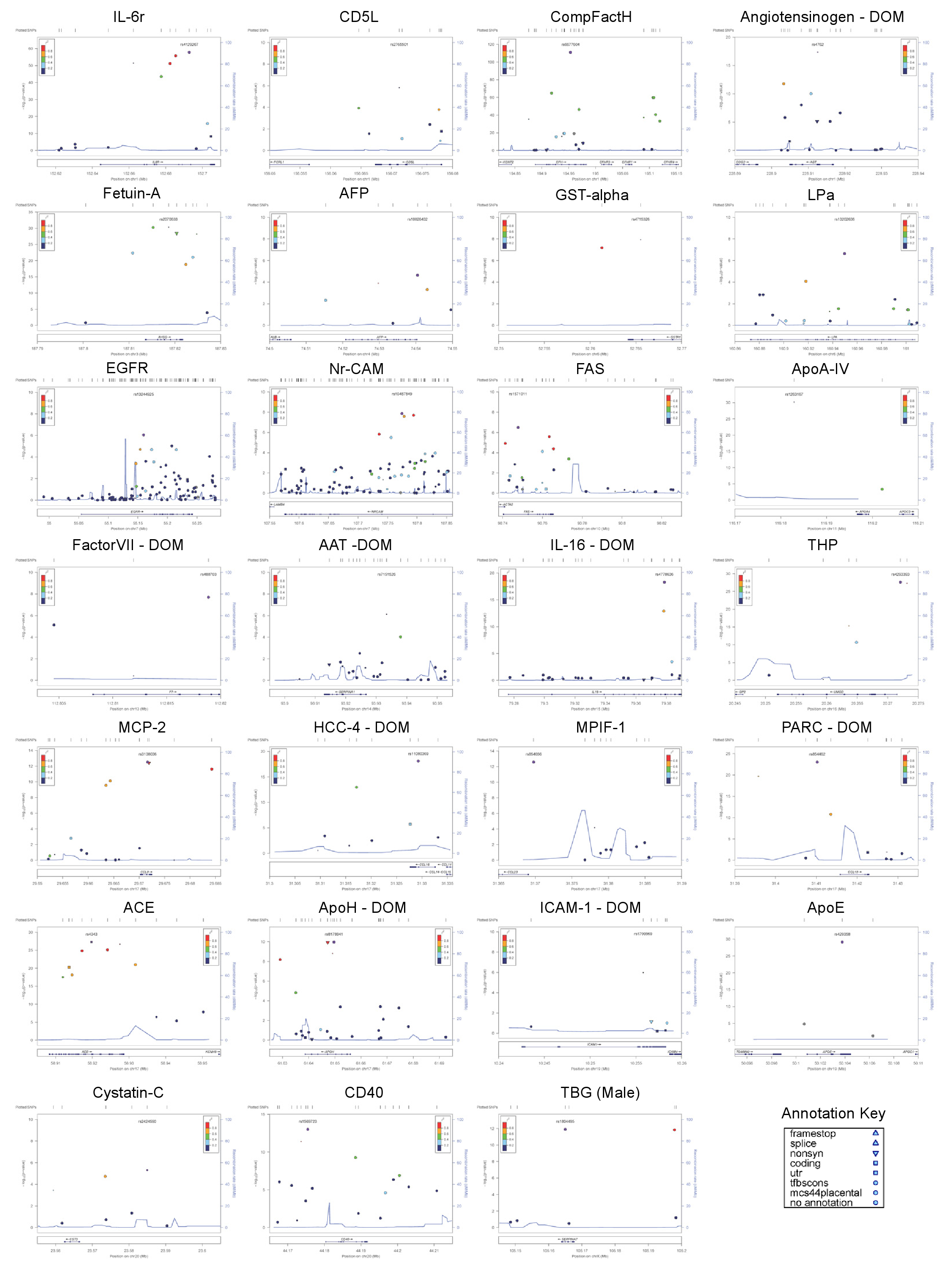

Supplement: Figure S1 — Zoomed view of association results. All association results between 28 gene-protein pairs from analyses were shown using LocusZoom (http://csg.sph.umich.edu/locuszoom/). In each panel, hg18 and HapMap Phase II CEU were used as Genome build and LD population. The panel for CompFactH shows the association of SNPs within CFH and CFHR1 together. Just for visualization purpose, the genetic mode tested for the most significant protein-SNP pair for each panel was selected and “DOM” represents a dominant genetic model. (TIF) [file pone.0070269.s001.tif]

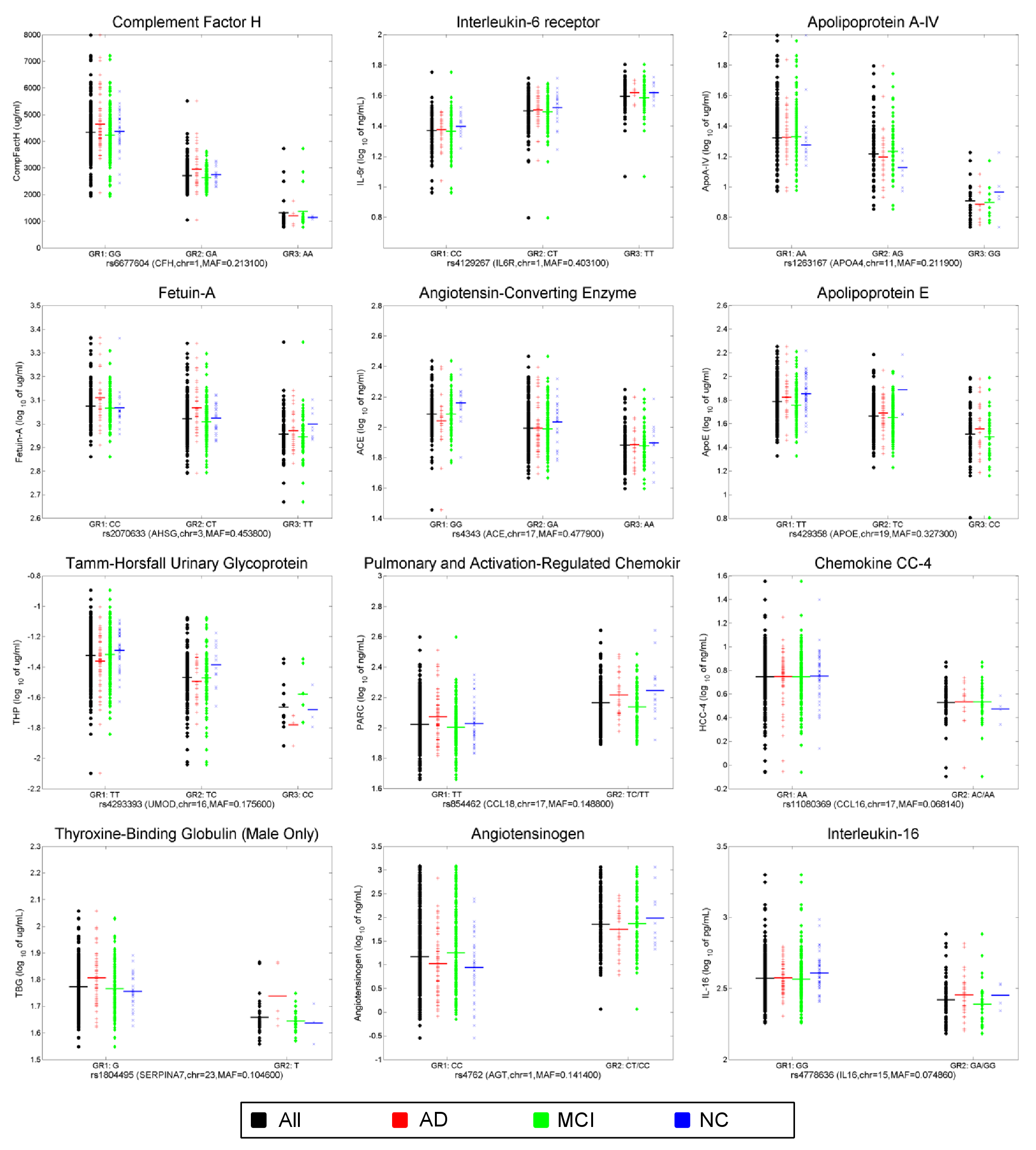

Supplement: Figure S2 — Scatter plots of top 12 associations in Table 3 for the ADNI cohort. Different colors represent different diagnoses (black - all, red - AD, green - MCI, blue - NC) and horizontal bars are the average protein levels within each group. Protein levels were adjusted for significant covariates (see Table S2) and APOE ε4 status except ApoE. For CompFactH, one association with rs6677604 was shown. (TIF) [file pone.0070269.s002.tif]
